# Low-Voltage 96 dB Snapshot CMOS Image Sensor with 4.5 nW Power Dissipation per Pixel

**DOI:** 10.3390/s120810067

**Published:** 2012-07-25

**Authors:** Arthur Spivak, Adam Teman, Alexander Belenky, Orly Yadid-Pecht, Alexander Fish

**Affiliations:** 1 The VLSI Systems Center, LPCAS, Ben-Gurion University, P.O.B. 653, Be'er-Sheva 84105, Israel; E-Mails: teman@ee.bgu.ac.il (A.T.); belenky@bgu.ac.il (A.B.); afish@bgu.ac.il (A.F.); 2 Department of Electrical and Computer Engineering, University of Calgary, Calgary, AB 13060, Canada; E-Mail: Orly.yadid.pecht@ucalgary.ca

**Keywords:** CMOS, image sensor, low power, snapshot, *SNR*, strong inversion, sub-threshold, wide dynamic range

## Abstract

Modern “smart” CMOS sensors have penetrated into various applications, such as surveillance systems, bio-medical applications, digital cameras, cellular phones and many others. Reducing the power of these sensors continuously challenges designers. In this paper, a low power global shutter CMOS image sensor with Wide Dynamic Range (*WDR*) ability is presented. This sensor features several power reduction techniques, including a dual voltage supply, a selective power down, transistors with different threshold voltages, a non-rationed logic, and a low voltage static memory. A combination of all these approaches has enabled the design of the low voltage “smart” image sensor, which is capable of reaching a remarkable dynamic range, while consuming very low power. The proposed power-saving solutions have allowed the maintenance of the standard architecture of the sensor, reducing both the time and the cost of the design. In order to maintain the image quality, a relation between the sensor performance and power has been analyzed and a mathematical model, describing the sensor Signal to Noise Ratio (*SNR*) and Dynamic Range (*DR*) as a function of the power supplies, is proposed. The described sensor was implemented in a 0.18 um CMOS process and successfully tested in the laboratory. An *SNR* of 48 dB and *DR* of 96 dB were achieved with a power dissipation of 4.5 nW per pixel.

## 1. Introduction

Advances in design techniques and fabrication technology have enabled the development of low-cost, multi-functional, low-power CMOS image sensors (CIS). Even though CMOS sensors naturally provide low power dissipation, their wide utilization in various portable battery-operated devices generates an increased demand for more aggressive power reduction techniques [1].

Throughout the past few years, numerous solutions for power reduction have been proposed. The most common approach for saving power is to scale down the supply voltages which bias the CIS. Scaling down the supply voltages reduces both dynamic and static power [2–5]. However, too aggressive supply reduction degrades the frame rate (*FR*), the dynamic range (*DR*) and the signal-to-noise ratio (*SNR*) of the imager. The regression of these figures of merit (FOMs) that is caused by the supply reduction is most pronounced if only a single voltage supply is used within the whole chip. In such a case, scaling down the power supply instantly affects all the blocks within the sensor, including those that designers might have preferred to leave unaffected.

The restrictions that are imposed by using a single power supply are mostly resolved by employing a dual supply approach [6]. According to this method, critical parts of the CIS, such as the pixel array and the analog processors, are biased with a high supply, whereas the periphery is powered by the lower supply. As a result, the designer can vary the power configuration of each block with greater flexibility. This additional degree of freedom, however, comes at the expense of the integration of a special interface that connects the blocks with different power supplies.

Possible solutions for power reduction can be applied at different abstraction levels: for example, power reduction at the algorithm level. Solutions that are applied at this level usually reduce the complexity of the calculations, which are needed for output signal processing. The complexity of calculations is eased by a reduction of the number of iterations for obtaining the final result [7] or by a controllable activation of some blocks. This occurs only when its input exceeds some predetermined threshold [8]. However, these solutions require adding processing circuitry, such as control or detection units. The additional circuitry not only dissipates power but also adds to the overall chip area. It is possible to alleviate the additional circuitry by reusing some units during the calculation. This technique is usually employed in analog to digital converters (ADCs), where it is possible to modify the operational amplifier feedback configuration by changing the connections between the feedback components [9]. Such circuitry reuse requires adding more control signals, which complicates the overall operational algorithm of the given CIS.

Other possible solutions have been proposed at the circuit and architecture levels. In many cases these solutions require substantial pixel structure modifications. For example, in [10] an additional source follower (*SF*) was accommodated within the pixel in order to increase the pixel swing under a low voltage supply. Such a readout chain is not typical in comparison to the majority of the pixels, in which each pixel employs only a single *SF* amplifier. Non-standard pixel structure is also presented in [11], where a power generating photo-diode was added to the regular one within each pixel.

Various low power solutions also affect the sensor periphery. For example, harvesting power [12] requires accommodating charge pumps and storage capacitors inside the sensor. Such changes are specific to this kind of solution and are not required in other low power solutions.

The goal of our research was to explore the possibility of designing a low power “smart” sensor using a standard architecture. In other words, we aimed to design a low power sensor by combining various design methodologies without modifying its typical architecture to achieve low power dissipation. It is important to note that a wide dynamic range sensor was chosen as a benchmark design in this research, since its typical architecture had been extensively researched by our group in the past [13]. The guidelines and techniques used in the presented design can therefore be easily incorporated in other CMOS sensors, regardless of their application.

The architecture of the presented sensor was adopted from our previous designs [13] with only slight modifications to meet the snapshot, low voltage, and low power requirements. Since we attempted to affect the sensor performance as little as possible, we used the dual supply voltage method. The dual supply solution presented in this study was improved as compared to that presented in [6]. However, though a dual supply voltage was used there too, the assignment of the low and the high power supplies was completely different. In that work, the high supply powered the pixel digital logic functions, whereas the lower supply powered the photodiode itself. This choice of power configuration certainly did not favor the photodiode performance and was imposed by relatively complex in-pixel logic architecture. Moreover, the pixel *SF* was realized using high threshold transistors, which reduced the output signal swing. Now on the other hand, we propose a pixel with substantially simplified architecture and improved *SF* amplifier. The pixel and the analog processing circuitry are powered by a single high power supply, whereas the lower supply powers the logic responsible for the *DR* extension. Furthermore, the presented solution includes the analysis of the dependence of *DR* and *SNR* on the power supply. We also present an effective procedure for finding the power supply configuration, upon which the sensor reaches the required *DR* and *SNR*, while consuming a minimal amount of power. The presented sensor was successfully designed, fabricated a 0.18 um CIS CMOS 1 poly 4 metal process and was tested in the lab, achieving 66 frames per second (fps), *SNR* of 48 dB, *DR* of 96 dB and a power dissipation of 4.5 nW per pixel.

The rest of the paper is organized as follows: Section 2 presents the system architecture and design considerations; Section 3 presents the configuration of power supply values along with measured experimental data; Section 4 concludes the paper.

## 2. System Architecture

### 2.1. Sensor Power Domains

**Figure 1.**
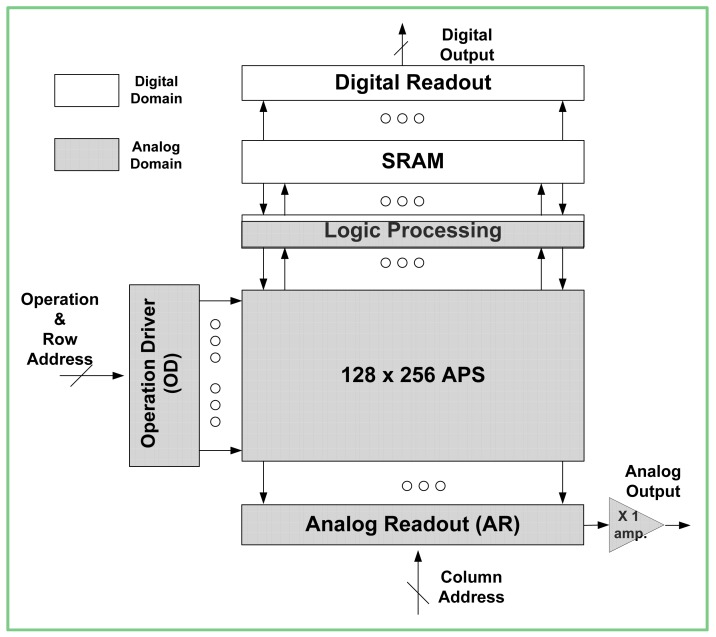
Block diagram of the sensor.

The sensor architecture is divided in accordance with the dual supply approach into two separate power domains: analog and digital. Each one of the power domains is biased by a separate power supply *AVDD* and *DVDD*, respectively (Figure 1). The darker areas are included in the analog power domain, which is biased with higher supply voltage, *AVDD*. The bright areas are included within the digital domain, powered by the lower supply voltage, *DVDD*. In this section we will discuss the design of blocks according to the power domain in which a certain block is found.

### 2.2. Analog Power Domain

**Figure 2.**
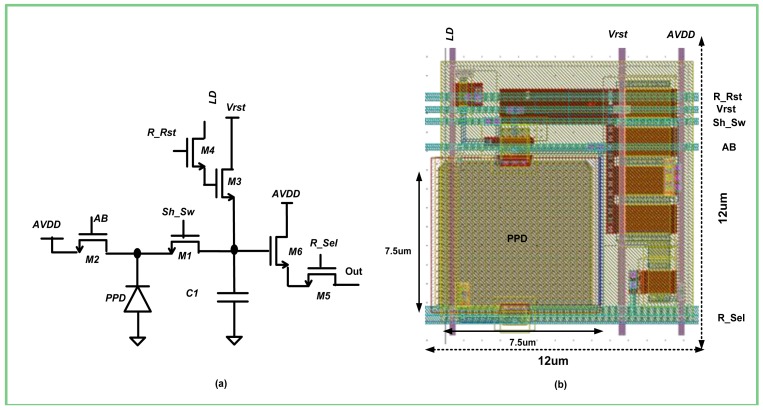
(**a**) Pixel schematic; (**b**) Pixel layout.

The analog power domain contains the pixel array and its periphery. An *Active Pixels Sensor (APS)* array is composed of 128 × 256 pixels (Figure 1). The photo-detecting element of each pixel is a *Pinned Photodiode* (*PPD*, Figure 2(a)). Herein, a photo-generated charge is transferred to integration capacitance *C_1_*, through transistor *M_1_*, controlled by the *Sh_Sw* signal. This charge transfer occurs at the selected time points throughout the integration. Since the *PPD* has a limited charge capacity, it can become flooded with charge before the last is passed on to *C_1_*. In such a case, if the charge is not supplied an alternative way, it will spill out uncontrollably from the *PPD* to the adjacent areas, causing the pixel to bloom. In order to prevent the blooming of the *PPD*, we included a separate transistor *M_2_*, which is controlled by the global signal *AB*. Thus, the overflowing charge, which cannot be transferred to *C_1_* integration capacitance, is dumped to the *AVDD* potential (Figure 2(a)). Moreover, by activating *AB* at the end of the frame, we ensured that the residual charge, which was not transferred to the integration capacitance *C_1_*, was drained out, thus preventing the image lag. It is important to note that both *AB* and *Sh_Sw* signals are always activated globally. Hence, the charge transfer throughout the array is applied to all the pixels simultaneously. Such simultaneous charge transfers enable the global (snapshot) operation of the presented sensor.

Minimal circuitry is required for the implementation of pixel functionality in order to reduce the area of the pixel. Besides the switches *M_1_* and *M_2_*, which control the direction of the charge flow, the pixel contains the “conditional reset” (*M_3_, M_4_*) circuitry that is similar to [13] and the typical *SF* amplifier (*M_5_, M_6_*), which is used for the pixel signal readout. The WDR operation is based on several saturation checks, which occur throughout the frame. During each saturation check, the *R_Rst* signal is raised, connecting the logic decision *LD* to the gate of the reset transistor *M_3_*. If the *LD* is high, *C_1_* is precharged to *V_rst_* potential; otherwise *C_1_* remains untouched. Once *C_1_* is not reset, it continues to receive a charge from the *PPD* without reset until the end of the frame. At the end of the frame, the charge, generated after the final saturation check, is transferred to *C_1_*. Then, by raising the *R_Sel* signal at the gate of *M_6_*, the charge is readout through the *SF* amplifier to the ADC. It should be noted that the integration capacitance *C_1_* is formed by parasitic capacitances of *M_1_, M_3_*, and *M_6_*, and, as such, does not occupy much space. By implementing the pixel with only six transistors and employing a part of their parasitic capacitances for the charge integration, we were able to implement a high density pixel layout, which occupied an area of 12 μm by 12 μm, 40% of which (7.5 μm by 7.5 μm) was occupied by the *PPD* (Figure 2(b)).

The proposed pixel employs transistors with different threshold voltages (Figure 2(a)) to achieve a better power performance tradeoff. The majority of transistors operate as switches, whose leakage should be minimized. Leakage minimization is usually achieved by high threshold transistors, which require relatively high gate voltage in order to ensure the full data pass. Therefore, in order to solve the tradeoff between the leakage and the level of the gate voltage, all the switches *M_1_*−*M_5_* were implemented with standard threshold transistors (SVT). The only transistor that was not operated as a switch is the input to the *SF*−*M_6_*. The threshold voltage of the *SF* input bounds the output pixel swing through the gate-source drop that was needed for current conduction: so we implemented *M_6_* with a low threshold transistor. The pixel swing extension made it possible to lower *AVDD* while keeping this transistor in the saturation region; hence the power reduction was achieved without degrading the *SF*'s gain.

By relying on the threshold of the transistors within the pixel, the values of the pixel power supply and the pixel control signals can be derived. There are two analog lines within the pixel: *V_rst_* is the bias that sets the reset level; *AVDD* is the power supply. Theoretically, *V_rst_* should be derived internally on the chip, using the bandgap reference. Since we did not implement it in the reported sensor, we powered the *V_rst_* line externally. Please note that, in both cases of the internal or external *V_rst_* generation, its value can be controlled by the designer. This control over *V_rst_* enables the flexible setting of the maximal and minimal pixel levels. By setting the appropriate bounds for the pixel signal, we can easily set the pixel swing needed for reaching the designated *SNR* and *DR*.

In order to set the pixel swing at the required level, while keeping the power as low as possible, the minimal values of the analog supply and the control signals should be calculated. Initially, we set the reset pixel level as:

(1) $$V_{\text{rst}}=V_{p}+V_{\text{sig}}$$

where *V_p_* is the potential that is required to completely deplete the *PPD* and *V_sig_* represents the desired pixel swing. Because it is essential to ensure that the target swing is not clamped by the pixel control signals, the logic ‘1a’ level for *R_Rst, Sh_Sw, AB*, and *R_sel* signals should be higher by the threshold voltage *V_th_* than *V_rst_* Equation (2). Since the level of these signals is the highest within the chip, the analog power supply is also given by Equation (2):

(2) $$‘1a’=\text{AVDD}=V_{R\_\text{Rst}}=V_{Sh\_Sw}=V_{AB}=V_{R\_\text{Sel}}=V_{\text{rst}}+V_{th}$$

The control over the snapshot array is performed by the *Operation Driver (OD)* block. The *OD* unit asserts a certain command to a selected row using a built-in row decoder. When a specific operation has to be applied simultaneously to the whole array, all the row decoder outputs are forced to logic ‘1a’. Since the logic ‘1a’ level is constant throughout all the units within the *OD*, this block is powered entirely by a supply whose value is equal to *AVDD*.

Another block that is related to the analog power domain is the *Analog Readout* (*AR*) unit. This unit is activated at the end of the frame. The final pixel values are read out row by row to the array of capacitors. Then, by means of the column decoder, this array of capacitors is sequentially sampled by the *X1 amp* (Figure 1). Similar to the *Operation Driver*, the *AR* is powered entirely by the analog supply.

Apparently the analog supply controls not just the pixel functionality, but also the operation of the readout chain from the *SF* through *AR* to the *Analog Output*; so the level of *AVDD* should be high enough to ensure low noise readout. A positive consequence of relatively high *AVDD* is decreasing the sensor noise floor by reduction of the sensitivity to process variations, thus minimizing the fixed pattern noise (FPN). For example, the switches within the pixel that are controlled by *AVDD* are operated in the linear region, hence their drain-source voltage in the steady state approaches zero and is independent of variations in the threshold voltage. In other circuits that do not operate as switches, for example *AR* or *X1 Amp*, using relatively high *AVDD* enables us to bias them in above threshold, *i.e.*, a strong inversion region. In such a case, although the dependence of the threshold voltage is not eliminated, it is minimized, since the dependency of the threshold is quadratic and not exponential as in the sub-threshold. In addition, the biases for analog readout chain have been selected carefully in order to solve the tradeoff between the minimization of the power that the specific circuit dissipates and the required speed at which this circuit should operate. Further power minimization in the analog readout chain was achieved by employing a separate power down signal, which disabled the relevant circuits as the pixels readout was over. We should note that in this design the bias voltages were also generated externally.

**Figure 3.**
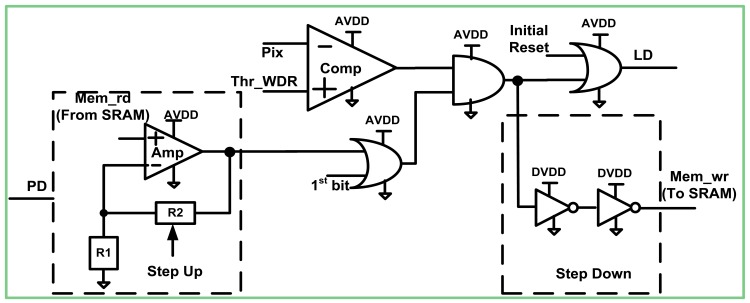
Schematic of the Logic Processing block.

All the blocks, discussed above, are powered by the analog supply, except for several externally generated bias lines. However, there is one unique block, the *Logic Processing* (*LPR*) unit (Figure 3) which relates to the analog power domain, but is powered by both power supplies. In this work we briefly describe its operation, but more detailed information can be found in [6]. The *LPR* unit is related to the analog domain, since most of its circuits are analog and not digital. However, there are several digital units that were included within the *LPR*. Incorporating digital blocks beside the analog blocks was necessary, since the *LPR* communicates with both the *APS* and the *SRAM*. The aim of the *LPR* block is to decide whether a certain pixel has to be reset or not at the current saturation check. The decision of the reset is received by *AND*ing the comparator *Comp* output with the result of the previous saturation check retrieved from the *SRAM: Mem_rd* signal. Please note that this output is in the digital domain. However, in the *LPR* unit, it takes part in switching of logic gates that are powered by the supply, the value of which equals *AVDD*, so a step up booster is inevitable. The step up function was implemented using a typical non-inverting amplifier *Amp* (Figure 3). We chose not to use the conventional level shifter structure, since we wanted to allow the logic to function with a sufficient speed under the widest possible range of *DVDD* values. The input of the conventional level shifter is composed out of native or high threshold NMOS transistors. Therefore, when the signal at their gate is far below the threshold voltage, they do not operate at all. Even when the input approaches the threshold, NMOS still operate relatively slow. On the other hand, the utilized non-inverting amplifier has input PMOS transistors and therefore can operate at ultra-low input voltages. From the simulations, we concluded that it can successfully elevate voltage signals as low as 0.3 V up to 2.5 V with a delay of couple of nanoseconds. In this way, we could examine the *DR* extension, when the digital circuits were found deeply in the weak inversion region.

The amplifier gain was obtained from the resistors ratio *R_2_* and *R_1_*, which were implemented with poly-silicon, characterized by extremely high resistance per square. Consequently, the area occupied by these resistors was minimized. Since we were interested in powering the digital domain with a variable *DVDD*, we made the gain variable, as well, by controlling the *R_2_* value. An important property of the *Amp* is that it can be cut off power by means of *PD* as soon as the current saturation check is over. In this way, the static power dissipation throughout the whole *LPR* block is minimized.

According to the presented logic scheme, a positive decision to reset the pixel is generated only if the pixel has been reset in the previous saturation check and the current pixel level (*Pix*) is below the predetermined threshold *Vth_WDR* in the current saturation check. In case the given saturation check is the first one within the frame, the *1st bit* signal is raised, making the reset decision dependent on the comparator output only. If the reset decision is positive, the line *LD* becomes high and activates the reset transistor within the pixel (Figure 2(a)). At the same time, the *Mem_wr* line goes high and the *SRAM* bit associated with that pixel is loaded with digital logic ‘1d’. In order to step down from possible “1a” value to “1d” value, which is appropriate for *SRAM*, we used two CMOS inverters, powered by the digital supply. In case the reset decision is negative, line *LD* remains low and the *SRAM* is fed with logic ‘0’. At the end of the frame, after the pixel was read out for the final A/D, a pixel is reset by asserting the *Initial Reset* signal, thus forcing the line *LD* to ‘1a’.

We can relate to the *LPR* block as a sort of connector between analog and digital domain. The *LPR* essentially separates the previously, discussed analog domain of the sensor and the further described digital domain.

### 2.3. Digital Power Domain

The digital power domain contains the *SRAM* and the *Digital Readout* (*DRD*) blocks (Figure 1). The *SRAM* stores the enumeration component of the *WDR* sensor. In order to reduce both the power consumption and the space, this on-chip *SRAM* is the same size as the pixel array, *i.e.*, 128 × 256 bits (32 kb), so each pixel is paired with a designated bit. Thus, the *SRAM* stores only the result of a single saturation check. The records of the previous saturation checks are stored in a large, off-chip memory. At each point in time, the execution of each pixel's reset is written to the on-chip *SRAM* and subsequently these values are added to the associated memory bits of the off-chip memory by means of the *Digital Readout*. As the imager asserts each row and performs a reset test, the previous reset value that is stored in the associated bit is checked to see if a reset operation is applicable, and then the new reset value is written to the temporary storage bit.

At the initiation of a reset evaluation time point, the entire 256 bit row is first read out to the *LPR* and subsequently written back from the *LPR*. Following this write-back, the new reset value is read out of the *SRAM* and sent to the external memory to be added to the previously accumulated number of resets for each pixel on the selected row.

Since the circuits in the digital domain are operated at low *DVDD* values, special attention was paid to the systems functionality under process variations. Circuits implemented with standard CMOS logic are non-rationed, and so they continue to function despite the process variations, albeit with varying performances. It is important to note that the functionality of the CMOS logic degrades substantially, as the circuit is biased deeply in sub-threshold or if the fan in exceeds a factor of 2 [14]. As a result, extensive simulations are needed in order to reveal the lowest voltage supplies, under which the non-rationed circuits are still feasible.

On the other hand, rationed circuits, such as the *SRAM* core, are sensitive to both global variations and local mismatch. At low voltages, the device drive strength can vary substantially, resulting in loss of functionality. *SRAM* operations at low voltages are generally limited by both read and write margins [15]. This *SRAM* block adopts some of the techniques described in [16,17] to enable robust functionality under process variations at the target operating voltage.

**Figure 4.**
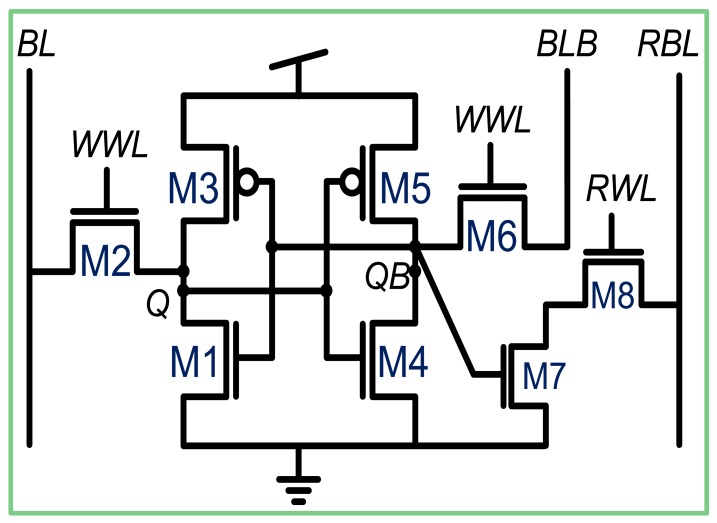
A standard 8T bitcell.

The general topology of the *SRAM* we used is the two-port 8-T bit-cell, which decouples the readout from the cell core (Figure 4). The structure employs a standard 6T bitcell core (*M1-M6*) with an additional pair of transistors (*M7-M8*) that comprise a readout buffer. Separate word lines (*WWL* and *RWL*) and bitlines (*WBL, WBLB* and *RBL*) are employed for write and read operations, respectively. In this way, during read operations, the bit-cell data is left undisturbed and therefore, the read margin is equivalent to the hold margin. However, the write margin remains a limitation for this type of cell, due to the rationed contention between the pull-up PMOS devices and the NMOS access devices during the write operations. In addition, for truly random accessible arrays, another situation occurs, known as “half select”, when only some of the bits in a row are written to. This presents a similar situation to a standard single-port read, as the bitlines of non-accessed cells are precharged.

In our topology, the half-select situation is irrelevant, because a full row is always accessed simultaneously. Therefore, the pull-down NMOS devices can be minimally sized for density. To address the write margin limitation, the length of the access transistors (*M_2_* and *M_6_*) is larger than the minimum length of the process technology in order to utilize the *Reverse Short Channel Effect* (RSCE) [18]. This strengthens the pull-down path during a write, ensuring that a write operation will be successful under 6σ variations.

Powering the *SRAM* with low *DVDD* values imposes certain limitations, not only on the *SRAM* core, but also on its periphery. Particularly, the sensing scheme was modified in order to make it possible to read out the memory contents throughout a wide range of *DVDD*. In the described design we used two sensing schemes. The first scheme was aimed at reading the cell values at relatively high supply value and as such employed typical sense amplifier [19]. The second scheme, on the other hand, was designed to operate under very low supply and was implemented by inverters. These two sensing schemes were activated selectively, according to the *DVDD* value, by an external control signal.

In addition to the sensing scheme, we also adapted the row and column access circuitry of the on-chip *SRAM* and the *DRD* units to low voltage operation. We used a serial access scheme by replacing the traditional row and column decoders with a shift register. This access scheme reduced the number of the control lines, area and power. The shift registers were designed to operate in wide range of *DVDD* power supply, including the sub-threshold region. Successful operation in the sub-threshold region was achieved by using typical NAND based flip-flops. These flip-flops provide the required robustness to the aggressive power supply scaling due to their unique architecture. The uniqueness of the NAND based flip-flop is that every output line of each NAND within the flip-flop sees high impedance, *i.e.*, the gate of the next NAND. This way every NAND gate can easily drive its output. Such feasibility towards the power supply change makes possible the aggressive supply scaling, thus resulting in the reduction of power consumption of *SRAM* and *DRD* units.

## 3. Power Consumption Minimization Analysis

In this section we present the procedure of choosing the values of both power supplies, so that the sensor will reach the desired *DR* and *SNR*, while consuming the minimal amount of power. When there are no power limitations, the desired values for *SNR* and *DR* are easy to set. However, when one attempts to reach these values while reducing power, completing the task is far more complex.

We began by setting the relation between the power supplies and the *SNR* and *DR*. The *SNR* depends on the ratio between the signal and its variance. Particularly, we were interested in finding the *SNR*, when the pixel is totally discharged, so that signal equals *V_sig_*. Throughout the whole frame, from the initial reset until the final readout, the pixel signal was controlled by *AVDD* only (Figure 2(a)). As a result, using Equation (1) and assuming that shot noise is the most prominent noise source, we got the following:

(3) $$\text{SNR}=20\log_{10}\frac{C_{1}V_{\text{sig}}}{\sqrt{qC_{1}V_{\text{sig}}}}=20\log_{10}\frac{C_{1}(\text{AVDD}-V_{th}-V_{p})}{\sqrt{qC_{1}(\text{AVDD}-V_{th}-V_{p})}}=20\log_{10}\sqrt{\frac{C_{1}(\text{AVDD}-V_{th}-V_{p})}{q}}$$

where *q* is the elementary electron charge.

The dynamic range of a pixel is comprised of two components: the intrinsic dynamic range *DR_intr_* and the dynamic range extension factor *DRF* Equation (4) [20]:

(4) $$DR=DR_{\mathrm{int}r}+\text{DRF}$$

The intrinsic dynamic range *DR_intr_* is given by the ratio between the maximum charge that the pixel can collect during the frame and the charge induced by the reset (KTC) noise:

(5) $$DR_{\mathrm{int}r}=20\log_{10}\frac{C_{1}V_{\text{sig}}}{\sqrt{2kTC_{1}}}=20\log_{10}[(\text{AVDD}-V_{p}-V_{th})\sqrt{\frac{C_{1}}{2kT}}]$$

The *DRF* component indicates how much the *DR* is to be increased. In this case, it is given by the ratio of the maximal and the minimal integration times: *t_int_max_, t_int_min_*, respectively Equation (6):

(6) $$\text{DRF}=20\log\frac{t_{\mathrm{int}\_\max}}{t_{\mathrm{int}\_\min}}$$

The minimal integration time essentially sets the *DRF*, since the maximal integration time always equals the frame time, which is constant. According to the algorithm that was used, each generated *WDR* bit should be written to the memory and then output off the chip. These bits are produced sequentially, thus, in order to prevent the loss of information, the generation of the subsequent bit should not be initiated before the previously generated bit is read out. Hence, the time that is required for producing and reading out a single bit bounds the minimal integration time of the given sensor. Consequently, the *DRF* depends on the speed of the operation of the *SRAM* and the *DRD* units. Since both of these units are associated with the digital domain, they are fully controlled by *DVDD*. It can be concluded that the analog supply affects both the *SNR* and *DR*, whereas the digital supply affects only the dynamic range extension.

By relying on the derived relation between the sensor performance and the values of the power supplies, we can also analyze how to scale them down so the sensor operates at the optimal point at which it reaches the designated performance and consumes a minimal amount of power. It can be observed that aggressive *AVDD* scaling is not possible, since this affects both the *SNR* and *DR*. Since the analog supply scaling in this case has too adverse an effect on the sensor performance, the only candidate that remains for this is the digital supply. Hence, our approach is to lower the *AVDD* minimally and substantially scale down the *DVDD*. This way, the sensor performance is expected to stay almost unchanged, while the power will be reduced remarkably.

Why is it possible to reduce *DVDD* without degrading the sensor performance? The proposed scaling is possible because the operation of digital blocks under high digital supply is often redundant in the sense of functionality and speed. The functionality depends upon two conditions: the first is that the block recognizes correctly the input levels of ‘1d’ and ‘0’ and the second is that its output is recognized appropriately by the next digital block. These two conditions are easily met for a wide range of the supply values, especially when using non-rationed logic, which can successfully operate even in the sub-threshold region. The main question is whether the certain non-rationed gate will operate with a sufficient speed if its power supply is lowered. If the supply is reduced too much, the gate delay will certainly exceed the required period of propagation. However, as the power supply increases, the delay drops. Consequently, the boundary power supply is the one that reduces the delay of the critical path to the designated value. From that value and on, any increase in the power supply becomes redundant and unnecessary.

In this case, after the *DVDD* exceeds the threshold voltage of the transistors used in the digital circuits, the delay caused by any digital block almost saturates. This means that, by further increasing of the *DVDD*, the system will show no improvement in its speed of operation. For example, the critical path delay should be 1clock cycle, which equals 25 ns. From the system point of view, there is no difference if the actual delay is 20 ns or 1 ns, under either a nominal or a scaled power supply, respectively. Therefore, it can be concluded that scaling down the *DVDD* towards the near threshold values will have no adverse effect on either the functionality or on the operation speed of the digital part of the sensor. We had verified the described assumptions regarding the *DVDD* scaling in numerous simulations. From these simulations we found that the functionality and the speed of the digital part could be preserved under a *DVDD* supply of 0.6 V, which is substantially lower than the nominal value of 1.8 V (Table 1). Please take into consideration that this value was obtained by taking into account the effects of both local and global variations. Because most of the power consumed by the digital circuits is dynamic with a quadratic dependence on the supply voltage, and nearly half of the sensor power has been contributed by the digital domain, the proposed aggressive digital supply scaling is expected to result in a substantial power reduction. It is very important to note that such scaling of the digital supply, which does not have much effect on the sensor performance, was made possible only due to the dual supply approach, which almost completely separated (aside from the *LPR*) the analog and digital power domains.

We would like to demonstrate the effects of power scaling on the sensor performance by analyzing three different test cases: *C1, C2*, and *C3* (Table 1). In each case, the sensor has a different supply configuration and, as such, reaches different *DR, SNR* and consumes a different amount of power. In fact, when two out of three parameters are set, it is easy to find the third parameter using either the derived relations Equations (1–6) or simulation software.

In the first test case *C1* we wanted to reach a *SNR* of 40 dB, while dissipating a minimal amount of power. Using Equation (5), we calculated the required *V_sig_*. Afterwards, using Equations (1,2), we obtained the *AVDD* value of 1.65 V (Table 1). The *DVDD* was lowered to its minimum (0 V), since no DR extension was required. In this case the anticipated DR equals 47 dB only. The requirements for the second test case *C2* were to achieve a *SNR* and *DR* of 50 dB and 98 dB, respectively. The analog supply value of 2.45 V was derived similarly to the first case, whereas the digital supply was set to its nominally highest value (1.8 V). In this way we aspired to test the maximal effect of the further *DVDD* scaling, which had been undertaken in the third test case *C3*. In that case the goal was to achieve an image quality comparable to the second case, but with much less power. If we were to convert the *SNR* of *C1* into image quality, we realized that its resolution exceeds 8 bits, so in *C3* we decided to limit the image resolution to 8 bits, therefore, using Equation (3), we observed that the *AVDD* should be lowered to 2.2 V. The DR extension in *C1* exceeds 6 bits; in *C3* it was lowered to 6 bits, resulting the overall DR of 96 dB. From the simulations, we concluded that the minimal *DVDD* that is necessary to reach the required performance is 0.6 V. We chose the *C3* point as the designated operation point of our sensor, since we felt it would successfully combine the power and the image quality. However, the final power configuration can vary substantially according to the preferences of a particular designer.

## 4. Experimental Results

### 4.1. Test Chip Measurements

**Figure 5.**
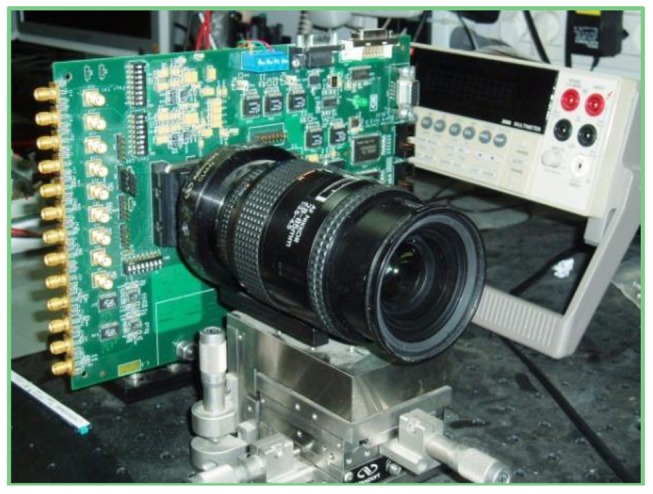
Sensor Test Board.

The presented 128 × 256 sensor was successfully implemented in a 0.18 μm CIS process. The fabricated chip was mounted on a test board (Figure 5), which powered the chip supplies and controlled the logic functions. Digital outputs of the sensor were scanned to the on-board static memory, while the analog output was fed to the on board pipelined A/D converter. PC software was used to image the captured scene. Various experiments were conducted to test a single pixel and the whole system performance. The final goal of the experiments was to verify the sensor functionality and its power consumption with variable power supplies. The measured results were in full agreement with the theoretical assessments; hence, they proved the feasibility of the proposed supply scaling and other low voltage design techniques implemented in the reported imager.

### 4.2. Pixel SF

The readout of an active pixel is performed through the *SF* amplifier. In order to test the *SF* performance, we shorted its input in some of the pixels to an electric contact. This way, we could determine exactly the input to the in-pixel amplifier and, by measuring the output, discover the offset and the gain of the implemented *SF*. According to the proposed design, this amplifier uses transistors with low and standard threshold voltage.

**Figure 6.**
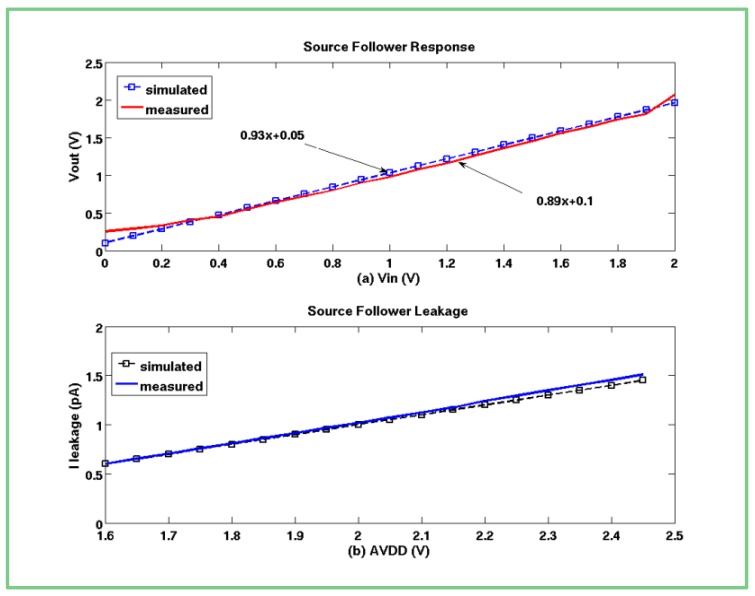
(**a**) SF Response; (**b**) SF Leakage.

The advantage of using a low voltage transistor as the input to the *SF* (*M_6_* in Figure 2(a)) can be clearly seen in Figure 6(a), where the designed *SF* reaches a swing of 2 V, demonstrating good linearity in both curves, representing the simulated and measured results, respectively. Moreover, the low threshold value also results in a larger transconductance factor *g_m_*, so that the *SF* gain remarkably exceeds the traditional gain of 0.78. The simulated gain is somewhat higher than the measured gain, which can be explained by the deviations in the model parameters between the simulated and fabricated input transistor.

A leakage current from an unselected pixel can cause a substantial crosstalk between the pixels that share the same column bus. A low threshold transistor can cause such a leakage through its terminals, since it becomes inverted at nearly zero gate-source voltage drop. Therefore, it was essential to verify that the row select switch (*M_5_* in Figure 2(a)), implemented with standard threshold transistor, presented very high impedance, and effectively disconnected the relevant pixel output from the column bus. The measured leakage current was substantially close to that predicted and had not exceeded 1.5 pA (Figure 6(b)). Since the pixel row conversion time was relatively short, the integrated on a sample capacitance leakage current did not affect the originally sampled pixel value, proving the feasibility of the proposed combination of transistors with different threshold voltages.

### 4.3. DR, SNR, and Power Measurements

**Figure 7.**
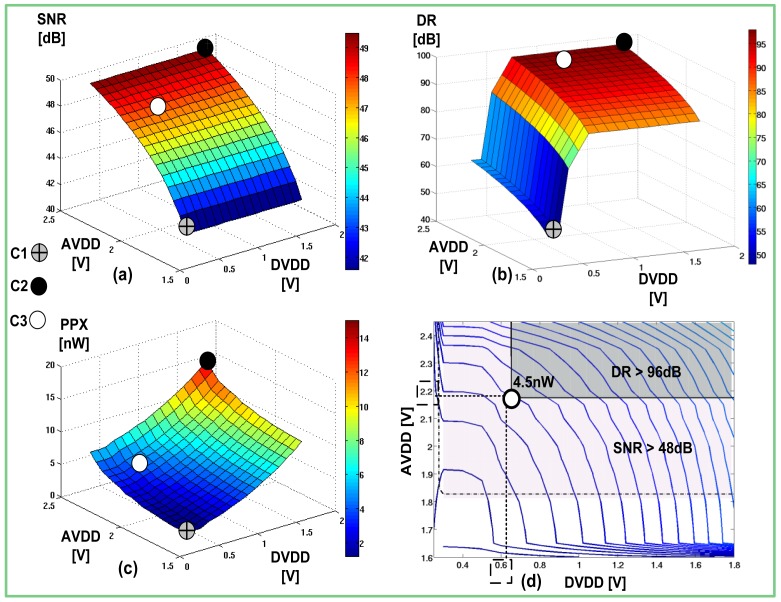
(**a**) SNR *vs.* AVDD *vs.* DVDD; (**b**) DR *vs.* AVDD *vs.* DVDD; (**c**) PPX *vs.* AVDD *vs.* DVDD; (**d**) Contour map.

We conducted numerous measurements of the sensor performance, while changing its power supplies. The sensor's *SNR* and *DR* were measured from the captured image using PC software, while the power consumption was derived by relying on the measurements of the current flowing through the *AVDD* and *DVDD* ports. During these measurements we changed the supplies' values separately in order to make it possible to represent the measured *SNR, DR* and power as functions of both power supplies. At the end of the measurements, we arranged the obtained data into 3D plots (Figure 7(a–c)), which effectively demonstrated the dependence of the measured parameters *vs.* the sensor supplies.

The theoretically assessed marginal values of *SNR* and *DR* were verified successfully in the measurements (Figure 7(a,b)). In order to facilitate the comparison of the power consumption characteristic with other sensors reported in the literature, we normalized the measured power by the number of pixels within the sensor array, obtaining power per pixel (*PPX*) (Figure 7(c)).

On these graphs we marked three different cases: *C1, C2*, and *C3*. Each point is located at different coordinates, defined by the values of *AVDD* and *DVDD*. *C1* and *C2* represent the boundary points with minimal and maximal power consumption. As anticipated, the first point is associated with minimal power consumption and the worst performance. On the other hand, point *C2* is associated with best image quality, but also with the highest power dissipation.

It is interesting to observe how the *SNR, DR* and *PPX* vary with the change of the power supplies. The *SNR* curve changes according to the square root of the analog power supply, regardless of the *DVDD*. On the other hand, the *DR* exhibits a more complicated behavior. In the direction of digital supply, it starts to grow rapidly between 0.3 V and 0.4 V, according to the exponential decrease in the delays of digital circuits that operate in the sub-threshold region. Gradually, the rate of change drops and finally becomes saturated as the logic circuits enter the strong inversion region at 0.6 V. A positive change of the *AVDD*, of course, increases the *DR*, but since this supply affects only the intrinsic component, its influence is remarkably smaller than that of the *DVDD*. Pixel power grows non-linearly in both directions, but, nonetheless, the influence of digital supply is somewhat more pronounced than the influence of the analog one, due to the quadratic dependence of the dissipated power on the supply.

With reference to the designated performance, neither *C1* nor *C2* can be selected to be the operating point of the sensor. At point *C1*, the image is poor, whereas at point *C2*, too much power is wasted. The optimal point *C3* can be found easily by using a contour map (Figure 7(d)). This figure depicts three contour maps: (1) presents the *PPX* levels; (2) presents the contour of designated *SNR* 48 dB, and (3) represents the contour of the designated *DR* of 96 dB. The common area defined by these three contours represents the region where all the requirements are met. Consequently, the optimal point is found at the boundary of the common area, where the *PPX* is minimal (4.5 nW). The coordinates of this point are the optimal values of the power supplies (0.65 V, 2.2 V). The measured values comply with the theoretical ones, listed in Table 1, by more than 92%.

**Figure 8.**
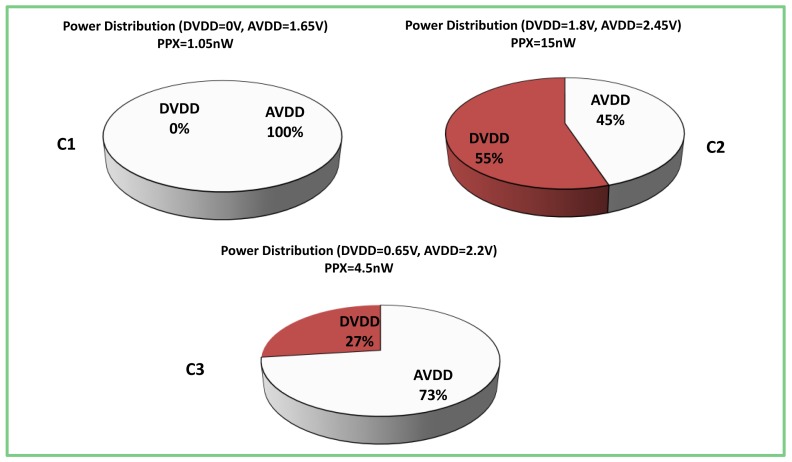
Power Distribution at Points C1, C2, and C3.

The proposed scaling of the sensor supplies substantially affects the power distribution (Figure 8). At point *C1*, the digital part almost does not consume power, thus the majority of it is dissipated within the analog domain. At point *C2*, which was the traditional operation point used before that presented in this article analysis; most of the power is divided almost equally between the *AVDD* and the *DVDD*. The effect of aggressive scaling of the digital supply can be clearly seen at point *C3*, where approximately ¾ of the overall power is consumed by the *AVDD*.

Table 2 summarizes some of the figures of merit of the presented sensor. The value of each measured parameter was shifted according to the *DVDD* or the *AVDD*, so we can denote the range of change rather than a single value. For example, as the *AVDD* is leveled up from 1.65 V to 2.45 V, the *Pixel Swing* increases from 0.2 V to 1 V and the FPN decreases from 1.5% to 0.1%.

In Table 3 we present quantitative comparison of our work with some state of art works in the field of low power CMOS sensors. The *PPX* values were derived upon the data, reported in that specific work. In [21] an on-chip spatial filter with eased computational complexity was implemented. Nevertheless, since there was no power supply optimization, quite high amount of power was consumed even without an on-chip ADC. On the other hand, we can see that the lately reported designs [3,4,12], where the power supplies were carefully tuned consumed very low power even with an on-chip ADC. However, this ultra-low power dissipation was achieved by very aggressive supply scaling, which resulted in degraded performance, especially in sensor speed. Table 3 highlights the fact that our sensor, operating at very high frame rate, achieves a remarkable power performance even though one of its power supplies is relatively high. Therefore, we can conclude that the presented work does not fall beyond the state-of-the-art designs in the field and that proposed herein selective supply assignment in conjunction with scaling is effective method to design low power CMOS smart sensor.

### 4.4. Captured Images

**Figure 9.**
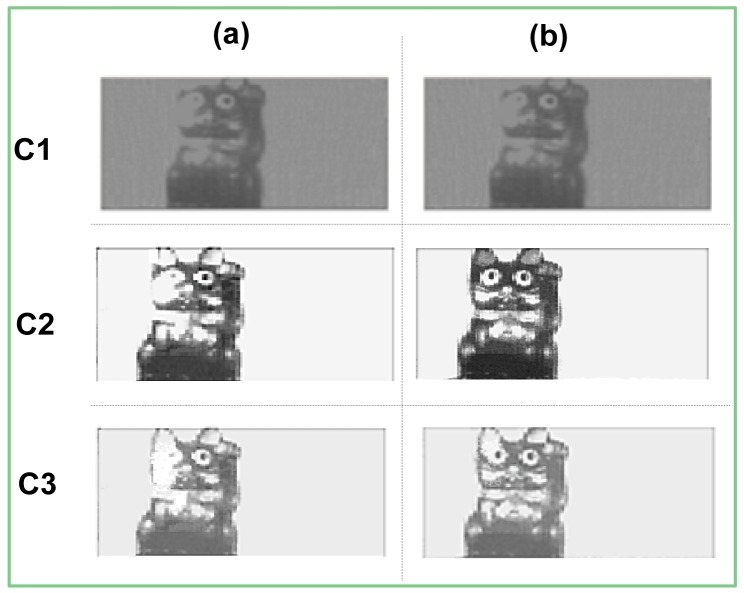
(**a**) Saturated images at points C1, C2, and C3; (**b**) WDR images at points C1, C2, and C3.

The captured images, Figure 9(a,b) demonstrate visually the difference between points *C1, C2*, and *C3*, respectively. The images found at the column (b) and (a) depict scenes captured with and without using the WDR extension, respectively.

At point *C1*, where the power supplies are at the minimal levels, the image quality is relatively poor, due to low pixel swing. The digital domain was operating too slowly to make possible any *DR* extension; therefore, the sensor elicited the same images with and without the WDR algorithm. Nevertheless, we are still able distinguish the image of the cat from its background.

As the sensor reaches the *C2* point, a substantial improvement in the quality of the captured images can be observed. Now, the *DR* extension can be clearly seen, when comparing between columns a and b, respectively. The saturated part of the cat is almost completely removed. Of course, the two images, captured at this point, have the highest *SNR* and *DR* among the three presented test cases.

At point *C3* both power supplies were scaled down. However, if the images captured at point *C3* to images at point *C2* are compared, it is hard to tell the difference immediately. This is logical since the *DR* and *SNR* values in these points are almost the same. However, upon closer examination, the differences can still be determined, especially in the *DR* extension, which is somewhat lower at point *C3*. Though most of the saturation is removed there, the inner areas of the cat ears are still saturated. However, taking into account that in point *C3* the power consumption is lower than in point *C2* (Table 3) by a factor of three, we conclude that the slight sacrifice in image quality is paid off by the remarkable power reduction.

## 5. Summary

We have presented a Low-Voltage Snapshot Wide Dynamic Range CMOS Image Sensor. A prototype of 128 × 256 pixels was fabricated using the 1-poly 4-metal CIS standard 0.18 μm process and was successfully tested. The proposed imager performs snapshot image acquisition and offers a linear increase in the dynamic range. The sensor was designed using a dual supply approach, according to which the sensor circuitry was divided into analog and digital domains; each domain was powered by a different supply. Such separation enabled an aggressive scaling of one of the power supplies, a *DVDD* in our case, without affecting the other. Thus, the performance parameters, which were independent of the scaled power supply, did not change. The effect on the parameters, which depended on the *DVDD*, was minimized by keeping it high enough to enable a sufficient robustness and speed of operation of blocks within the digital power domain. On the other hand, the value of the *AVDD* was merely reduced to keep the designated sensor performance. Further power optimization was achieved by the integration between low and medium threshold transistors, leakage reduction, and low voltage SRAM incorporation. By using the proposed dual supply approach and power reduction steps described above, we succeeded in designing a low power CMOS sensor with a standard architecture, while obtaining remarkable sensor performance and image quality.

## Figures and Tables

**Table 1.** AVDD and DVDD values to reach Minimal, Nominal and Optimal SNR and DR.

	**C1 SNR = 40 dB, DR = 47 dB**	**C2 SNR = 50 dB, DR = 98 dB**	**C3 SNR = 48 dB, DR = 96 dB**
AVDD	1.65 V	2.45 V	2.2 V
DVDD	0 V	1.8 V	0.6 V

**Table 2.** Measured sensor attributes.

**Parameter**	**Measured Value**		

AVDD	1.65 V–2.45 V		
DVDD	0 V–1.8 V		
Array Size	128 (rows) × 256 (columns)		
Photo-sensing Area	7.5 um × 7.5 um		
Maximal Conv. Gain	84 uV/e		
Pixel Swing	0.2 V–1 V		
Dark Current	0.09 fA		

**Parameter**	**C1**	**C2**	**C3**

FPN	1.5%	0.1%	0.5%
DR	47 dB	98 dB	96 dB
SNR	40 dB	50 dB	48 dB
PPX	1.05 nW	15 nW	4.5 nW

**Table 3.** Comparison of the presented design and other related works in the field.

**Parameter**	[21]	[3]	[4]	[12]	This work
**Technology**	0.5 um	0.13 um	0.13 um	0.35 um	0.18 um
**Power Supplies**	-	0.5 V	0.75 V, 1.25 V	1.35 V	2.2 V, 0.65 V
**Array Size**	33 × 25	128 × 128	128 × 128	128 × 96	128 × 256
**Frame Rate**	0–10^4^ fps	8.5 fps	15 fps	9.6 fps	60 fps
**On-chip ADC**	no	yes	yes	yes	no
**PPX**	300 nW	0.073 nW	0.098 nW	0.8 nW	4.5 nW (C3)

## References

[1] Fish A., Yadid-Pecht O. Low-Power Smart CMOS Image Sensors.

[2] Chae Y., Cheon J., Lim S., Lee D., Kwon M., Yoo K., Jung W., Lee D.-H., Ham S., Han G. A 2.1Mpixel 120frame/s CMOS Image Sensor with Column-parallel ΔΣ ADC Architecture.

[3] Hanson S., Zhi Y.F., Blaauw D., Sylvester D. (2010). A 0.5 V sub-microwatt CMOS image sensor with pulse-width modulation read-out. J. IEEE Solid-State Circuit..

[4] Cho K., Lee D., Lee J., Han G. (2010). Sub-1-V CMOS image sensor using time-based readout circuit. IEEE Trans. Electron Devices.

[5] Ignjatovic Z., Maricic D., Bocko M.F. (2012). Low power, high dynamic range CMOS image sensor employing pixel-level oversampling ΣΔ analog-to-digital conversion. IEEE Sens. J..

[6] Belenky A., Fish A., Spivak A., Yadid-Pecht O. (2009). A snapshot CMOS image sensor with extended dynamic range. IEEE Sens. J..

[7] Lim S., Lee J., Kim D., Han G. (2009). A High-Speed CMOS image sensor with column-parallel two-step single-slope ADCs. IEEE Trans. Electron Devices.

[8] Dongsoo K.D., Fu Z.-m., Joon H.P., Culurciello E. (2009). A 1-mW CMOS temporal-difference AER sensor for wireless sensor networks. IEEE Trans. Electron Devices.

[9] Lin J.-F., Chang S.-J., Chiu C.-F., Tsai H.-H., Wang J.-J. (2009). Low-Power and wide-bandwidth cyclic ADC with capacitor and opamp reuse techniques for CMOS image sensor application. IEEE Sens. J..

[10] Xu C., Ki W.-H., Chan M. (2002). A low-voltage CMOS Complementary Active Pixel Sensor (CAPS) fabricated using a 0.25 μm CMOS technology. IEEE Electron. Device Lett..

[11] Fish A., Hamami S., Yadid-Pecht O. (2006). CMOS Image Sensors with Self-Powered Generation Capability. IEEE Trans. Circuit. Syst-II: Express Briefs.

[12] Tang F., Bermak A. (2012). An 84 pW/Frame per pixel current-mode CMOS image sensor with energy harvesting capability. IEEE Sens. J..

[13] Yadid-Pecht O., Belenky A. (2003). In-pixel autoexposure CMOS APS. IEEE J. Solid-State Circuit..

[14] Wang A., Calhoun H.B., Chandrakasan A. (2006). Sub-Threshold Design for Ultra Low-Power Systems.

[15] Calhoun B.H., Chandrakasan A.P. (2007). A 256-kb 65-nm sub-threshold SRAM design for ultra-low-voltage operation. IEEE J. Solid State Circuit..

[16] Kim T.-H., Liu J., Kim C.H. An 8T Subthreshold SRAM Cell Utilizing Reverse Short Channel Effect for Write Margin and Read Performance Improvement.

[17] Verma N., Chandrakasan A.P. (2008). A 256 kb 65 nm 8T subthreshold SRAM employing sense-amplifier redundancy. IEEE J. Solid-State Circuit..

[18] Kim T.H., Keane J., Eom H., Kim C.H. (2007). Utilizing Reverse Short-Channel Effect for Optimal Subthreshold Circuit Design. IEEE Trans. Very Large Scale Integr..

[19] Nambu H., Kanetani K., Yamasaki K., Higeta K., Usami M., Kusunoki T., Yamaguchi K., Homma N. (1998). A 1.8-ns access, 550-MHz, 4.5-Mb CMOS SRAM. IEEE J. Solid-State Circuit..

[20] Spivak A., Belenky A., Fish A., Yadid-Pecht O. (2009). Wide-Dynamic-Range CMOS image sensors-comparative performance analysis. IEEE Trans. Electron. Devices.

[21] Lin Z., Hoffman M.W., Schemm N., Leon-Salas W.D., Balkir S. (2008). A CMOS image sensor for multi-level focal plane image decomposition. IEEE Trans. Circuit. Syst..

